# Our Impatient Bacteriologist

**Published:** 1911-11

**Authors:** 


### Our Impatient Bacteriologist

Major William G. Bissell, City Bacteriologist of Buffalo, in the first test of the filters installed in the public schools has found them unsatisfactory. Representatives of the manufacturers claim that the test was unfair as they had no notice of the date at which the test was to be made and no chance to prepare for it. This claim is admitted. The water, by the way, had a higher bacterial count after than before filtration. We would suggest that sufficient notice should be given those interested in the filters so that they could have them sterilized in advance of the test and that the water pipes should be tapped so as to run a supply of boiled water through them into the filters. The result would then be available as an advertisement. As to the children of three grammar schools and three high schools that drink the water in the mean time—well, that is another matter.

